# Characterization of *Sus scrofa* Small Non-Coding RNAs Present in Both Female and Male Gonads

**DOI:** 10.1371/journal.pone.0113249

**Published:** 2014-11-21

**Authors:** Dorota Kowalczykiewicz, Aleksandra Świercz, Luiza Handschuh, Katarzyna Leśniak, Marek Figlerowicz, Jan Wrzesinski

**Affiliations:** 1 Institute of Bioorganic Chemistry, Polish Academy of Sciences, Poznań, Poland; 2 Institute of Computing Science, Poznań University of Technology, Poznań, Poland; 3 Department of Hematology, Poznań University of Medical Sciences, Poznań, Poland; CNRS UMR7622 & University Paris 6 Pierre-et-Marie-Curie, France

## Abstract

Small non-coding RNAs (sncRNAs) are indispensable for proper germ cell development, emphasizing the need for greater elucidation of the mechanisms of germline development and regulation of this process by sncRNAs. We used deep sequencing to characterize three families of small non-coding RNAs (piRNAs, miRNAs, and tRFs) present in *Sus scrofa* gonads and focused on the small RNA fraction present in both male and female gonads. Although similar numbers of reads were obtained from both types of gonads, the number of unique RNA sequences in the ovaries was several times lower. Of the sequences detected in the testes, 2.6% of piRNAs, 9% of miRNAs, and 10% of tRFs were also present in the ovaries. Notably, the majority of the shared piRNAs mapped to ribosomal RNAs and were derived from clustered loci. In addition, the most abundant miRNAs present in the ovaries and testes are conserved and are involved in many biological processes such as the regulation of homeobox genes, the control of cell proliferation, and carcinogenesis. Unexpectedly, we detected a novel sncRNA type, the tRFs, which are 30–36-nt RNA fragments derived from tRNA molecules, in gonads. Analysis of *S. scrofa* piRNAs show that testes specific piRNAs are biased for 5′ uracil but both testes and ovaries specific piRNAs are not biased for adenine at the 10^th^ nucleotide position. These observations indicate that adult porcine piRNAs are predominantly produced by a primary processing pathway or other mechanisms and secondary piRNAs generated by ping-pong mechanism are absent.

## Introduction

Three major families of small non-coding RNAs (sncRNAs) have been identified in eukaryotic cells: microRNAs (miRNAs), short interfering RNAs (siRNAs), and Piwi-interacting RNAs (piRNAs) [1],[2]. miRNAs, which are the best-described family, are RNA molecules 21–23 nucleotides (nt) in length that are encoded in the nuclear genome. In the human genome, up to 2,500 frequently polycistronic miRNA genes have been identified [3]. miRNA biogenesis is relatively well understood and involves the processing of the primary transcript by a set of nucleases [4]–[7]. The pri-miRNA, which forms a characteristic stem-loop structure, is processed in the nucleus by Drosha ribonuclease upon binding of the Dgcr8 protein. The resulting precursor miRNA (pre-miRNA) is exported to the cytoplasm and is cleaved by the Dicer endonuclease to produce the mature miRNA. The miRNA is incorporated into the RNA-induced silencing complex (RISC) formed by the Ago proteins. In the RISC complex, the miRNA serves as a probe that recognizes the complementary mRNA and inhibits its expression [7]. miRNAs play a significant role in gametogenesis. Mutation of the murine Drosha and Dicer nucleases, which affect miRNA biogenesis, impacts the spermatogenesis pathway, leading to complete testicular degeneration [8]–[10]. Mutation of Dicer arrests oogenesis at the meiosis I step [10],[11]. Furthermore, miRNAs such as let-7 and members of the miR-290 cluster influence the early stages of gametogenesis, namely, primordial germ cell specification and migration [12],[13].

In contrast to miRNAs, endogenous siRNAs (∼22 nt in length) are produced from long dsRNAs by Dicer [14],[15]. Similar to miRNAs, siRNAs associate with the Ago proteins family to form RISC complexes, which bind to the target mRNAs thus destroying them. However, while siRNAs typically form perfect duplexes with the target mRNAs and induce their cleavage, miRNAs inhibit the translation of target mRNAs and form imperfect duplexes with them [10]. Both siRNAs and miRNAs may play a role in epigenetic DNA modifications through a process called RNA-induced transcriptional silencing or RNA-induced DNA modification. It is estimated that the expression over 60% of all genes is regulated by miRNAs or siRNAs [16]. The influence of endogenous siRNAs (endo-siRNAs) on gametogenesis has been studied in the nematode *C. elegans*. Mutations affecting the biogenesis of the germline-specific 22G and 26G siRNAs result in defects in spermatogenesis due to the failure of chromosome segregation during mitosis and meiosis [11],[17],[18]. In addition, the absence of RNA-dependent RNA polymerase, an enzyme important for the synthesis of the endo-siRNA precursor, results in severe defects during oogenesis and the production of defective oocytes [19]. The impact of endo-siRNAs on eukaryotic gametogenesis is less well recognized. Endo-siRNA production in the mouse germline cells was previously detected by deep sequencing [20].

The piRNAs are a different class of small non-coding RNAs and, in contrast to miRNAs and siRNAs, are found exclusively in the gonads. They have been detected in many organisms (from *Drosophila* to human) with diverse maternal and paternal reproductive systems [21]. piRNAs function in ribonucleoprotein complexes with Piwi proteins. Between two and four Piwi proteins have been identified in various organisms [21],[22]. Similar to other mammalian gonads, the porcine gonads encode three Piwi proteins, namely, Piwil1, Piwil2, and Piwil4 [23]. The slicer activity of the murine Piwil2 (Mili) proteins is potentially involved in the piRNA biogenesis. The change of the Piwil2 (Mili) catalytic triad DDH with a DAH motif generates a Piwil2^DAH^ mutant mice in which biogenesis of piRNA is affected during spermatogenesis. Additionally, in the case of a homozygous Piwil2^DAH^ mouse, piRNAs production is low and the number of piRNA is significantly reduced [24]. Furthermore, deletion of the murine *piwil4* gene affects spermatogenesis and causes massive degeneration of the spermatogonia [25]. Disturbances of the piRNA biogenesis pathway and the loss of Piwi protein expression in males typically result in infertility.

A new group of sRNAs, the tRFs, was recently discovered. tRFs are 30–36 nt fragments of tRNA [26]–[28]. The tRF family has been suggested to be involved in the stress response and in tumor suppression [27,29],[29].

Recent studies have demonstrated that porcine physiology pathology and reproduction is similar to that of human than that of other animals that are frequently used as research models, namely, nematodes (*C. elegans*), flies (*D. melanogaster*), and rodents such as mice (*M. musculus*) and rats (*R. norvegicus*). Therefore, the pig (*S. scrofa*) is increasingly used as a model system for various biological and biochemical studies [30]. An important advantage of this model system is the knowledge of the *S. scrofa* genome, which was determined by the Swine Genome Sequencing Consortium [31].

Here, we report the characteristics of the small RNA molecules (piRNAs, miRNAs and tRFs) detected in porcine gonads by deep sequencing. The main goal of our analysis was to determine if there is a pool of small RNAs that are present in both female and male gonads or if the small RNAs that occur in testes and ovaries significantly differ from each other.

## Materials and Methods

### Isolation of sncRNAs

For the Illumina deep sequencing, we used testes and ovaries collected from sexually mature 1 year old female and male pigs (*S. scrofa domestica*). Before dissecting each pair of ovaries, we evaluated the corresponding uterus to distinguish between young and multiparous females. The testes were collected from males of Line 990. Both female and males were obtained from the Experimental Station of The National Institute of Animal Production (Pawlowice, Poland) and were slaughtered under the supervision of a certified veterinary doctor. Immediately after slaughter, the tissues including the testes and ovaries were isolated, frozen in liquid nitrogen, and stored at −80°C until analysis. This study was conducted with the permission of the National Animal Experimentation Ethics Committee, Local Committee in Poznan (permission number 70/2008).

The sncRNAs were isolated using the mirVana miRNA isolation kit according to the manufacturer's instructions (Ambion). A total of 300 mg of tissue was briefly homogenized in 10 volumes of Lysis/Binding buffer. A 1/10 volume of miRNA homogenate additive was added, and the samples were incubated on ice for 10 min. Total RNA was extracted by adding an equal volume of acid-phenol:chloroform. RNA-containing aqueous phase was mixed with 1/3 volume of 100% ethanol, applied to a filter cartridge and centrifuged. The sncRNA fraction present in a filtrate was then mixed with 2/3 volume of 100% ethanol and purified using a second filter cartridge. Concentration of eluted snc RNAs was measured using a NanoDrop spectrophotometer. For Illumina sequencing, three independent isolations were performed from two adult, sexually mature, female and male porcine gonads, and the obtained sncRNA fractions were combined and used for further analysis.

### SncRNA library preparation

The sncRNA fractions extracted from porcine gonads (ovaries and testes) were subjected to 12% (w/v) denaturing PAGE (polyacrylamide gel electrophoresis) and the sncRNA fragments 18–40 nucleotides were isolated. In the next step RNAs were analyzed using a Bioanalyzer 2100 with a small RNA assay (Agilent). 1 *µ*g of each sncRNA sample was used to prepare a sequencing library with the TruSeq sncRNA Sample Prep Kit (Illumina), according to the TruSeq Small RNA Sample Preparation Guide. Briefly, sncRNAs were subsequently ligated with RNA 3′ and 5′ adapters (each ligation was carried out in 28°C for 1 h) and reversely transcribed using SuperScript III Reverse Transcriptase (Invitrogen) at 50°C for 1 h. The synthesized cDNA was then amplified using Phusion DNA Polymerase, indexed primers and 11 PCR cycles (98°C 10 s, 60°C 30 s, 72°C 15 s) and validated with High Sensitivity DNA Chip (Agilent). The amplified cDNA library was size-selected by electrophoresis (60 min, 145 V) in 6% Novex TBE PAGE Gel (Invitrogen). Fragments in the range of length 140–190 nt, corresponding to small RNAs (15–70 nt) with both adapters, were extracted from gel using Gel Breaker tubes (IST Engineering) and 3-h-incubation in water (RT, Rotator Mixer RM-Multi 1 (Starlab)). Then the libraries were purified on 5 µm filter tubes (IST Engineering) and concentrated by ethanol (100%, 3,25 vol.) and sodium acetate (3 M, 1/10 vol.) precipitation. After the second validation with High Sensitivity DNA Chip (Agilent) the libraries were quantified using Qubit fluorometer (Invitrogen). Separate peaks corresponding to the miRNA and piRNA fractions were observed in electropherograms of both testis- and ovary-derived libraries.

### Next-generation sequencing

Next-generation sequencing was performed using the Illumina Genome Analyzer IIx at the European Center of Bioinformatics and Genomics in Poznan. 10 pM of each library was mixed with other indexed sncRNA libraries and sequenced on a single-read flow cell, eight samples per lane, with the exception of the control lane intended for the Phix library (Illumina). Each library obtained from the testes and ovaries generated approximately 6 million of 72-nt-long reads. The adapter sequences were clipped from the raw sequences, allowing one mismatch in the adapter sequence. Over 97% of the reads were retrieved. The low-quality reads were filtered out, leaving only the sequences with a quality score greater than or equal to 30 for at least 80% of the nucleotides; approximately 4.5% of all reads were filtered out. Next, redundancies were removed by retaining only a single occurrence of each sequence (the number of occurrences of each sequence was noted). This step reduced the number of reads to 15.5% for the testis library and 3.5% for the ovary library. Each library was divided into two sets of reads, depending on their length: miRNAs (19–25 nt), piRNAs, and tRFs (26–40 nt). Two other sets originated from the intersection of the reads appearing in both the ovary and testes libraries. Thus, we finally obtained six sets of reads: miRNA, piRNA, and tRF sequences appearing in the testicular library and miRNA, piRNA, and tRF sequences appearing in the ovarian library. Adapter trimming, quality filtering, and redundancy removal were performed with the FASTX-Toolkit, and libraries were created using in-house scripts written in C++ and awk.

### The genome assembly

The sncRNAs were mapped to the swine genome (Illumina iGenomes, *S. scrofa* version 10.1) using SOAP [32], allowing up to one mismatch. Shorter sequences, miRNAs, were mapped with the ‘seed length’ set to 8, while longer, piRNAs and tRFs used ‘seed length’ set to 10. The SOAP determined each genomic position to which the read was mapped. More than 76% of reads could be mapped to the genome, and 70% of those reads mapped to unique locations. The genome was masked using RepeatMasker [33] to obtain genomic coordinates of repetitive elements, and this information was used to annotate the reads as tRNAs, rRNAs, snRNAs, scRNAs, and other classes of repeats. Sequences of the ovarian and testicular piRNA libraries were mapped to the *Sus scrofa* tRNA sequences (Genomic tRNA Database at http://gtrnadb.ucsc.edu) using Blastn. The reads containing CCA at the 3′ end (corresponding to tRNAs) were trimmed to discard the trinucleotide terminal sequences that do not occur in the genome and avoid additional bias. All sequences which were then aligned with up to one mismatch and were annotated as tRF.

### piRNA cluster analysis

A piRNA cluster was defined as a group of at least 80 piRNAs located not more than 2,500 nt away from each other on a chromosome. The piRNAs mapped to several locations in the genome, and each location was considered for cluster identification.

### Labeling of the 5′-ends of piRNAs

piRNAs with a length of 28 to 35 nt from the testes and ovaries were purified from 5–8 µg of low-molecular-weight RNA by excision of the corresponding gel slices from SYBR-gold-stained, 15% denaturing polyacrylamide gels. The piRNAs were recovered from the excised gel slices by elution with 0.3 M sodium acetate and 1 mM EDTA buffer. Approximately 150 ng of the piRNA fraction from both gonads was 5′-end-labeled using T4 polynucleotide kinase (Ambion) in the presence of [γ-^32^P] ATP (Hartmann Analytic), followed by dephosphorylation using calf intestinal phosphatase (Ambion). The labeled piRNAs with and without alkaline phosphatase treatment were separated on a 12% polyacrylamide-urea gel. Fujifilm FLA-5100 was used for radioactive imaging analysis.

### *In vitro* transcription

Two synthetic oligonucleotides were annealed to form a double-stranded template for transcription containing a T7 RNA polymerase promoter site and a piRNA sequence found in *S. scrofa*. To obtain a dsDNA template, both oligonucleotides were mixed in equimolar amounts to a final concentration of 50 µM each. The mixture was incubated at 100°C for 2 min, on ice for 5 min, and at 37°C for 5 min. The 63/729 transcript was obtained using the MEGAshortscript Kit, according to the manufacturer's instructions (Ambion). A solution of 2 mM guanosine was added to the reaction and incubated at 37°C for 4 h. The transcript band was excised from an 8% polyacrylamide-urea gel. The full-length RNA transcript was recovered from the excised gel slice by elution with 0.3 M sodium acetate and 1 mM EDTA buffer. A total of 75 ng of the 63/729 piRNA transcript was 5′-end-labeled using T4 polynucleotide kinase in the presence of [γ-^32^P] ATP and separated on a 12% polyacrylamide-urea gel. The labeled transcript was recovered from the gel by elution and used as a control in the β-elimination reaction.

### β-elimination reaction

The piRNA fractions from the gonads of adult animals were isolated and 5′-end-labeled using the KinaseMax Kit according to the manufacturer's instructions (Ambion). Sodium periodate treatment and β-elimination were performed. The sRNA samples were briefly incubated with 10 mM NaIO_4_ (Sigma) and tRNA (Sigma, final concentration 200 ng/µl) for 1 h on ice in the dark. For each sample, a control reaction was performed containing water instead of NaIO_4_. The RNA was precipitated with ethanol at −20°C overnight, and the precipitate was collected by centrifugation and dissolved in 60 µl of 1 M lysine, pH 8.5. The samples were incubated for 90 min at 45°C. Then, 20 µg of glycogen, ethanol, and sodium acetate were added, and the RNA was precipitated at −20°C overnight. The precipitate was collected by centrifugation and dissolved in denaturing gel loading buffer. The products of the β-elimination reaction were resolved by 12% denaturing polyacrylamide gel electrophoresis (PAGE). Fujifilm FLA-5100 was used for radioactive imaging analysis.

### Data depostion

*S. scrofa* sncRNA sequencing data have been deposited in the NCBI Gene Expression Omnibus (GEO) (http://www.ncbi.nlm. nih.gov/geo/) under accession no. GSE57414.

## Results and Discussion

### Characteristic of *S. scrofa* gametogenesis

Reproduction is one of the most important life processes ensuring the survival of animal species and many animal models involving pig are used to fully understand it. Pig gametogenesis starts from primordial germ cells (PGC) that colonize specific sites of the embryo giving rise to testis or ovary. Depending on the sex, the process of gonad formation is characterized by significant differences in the gene expression profiles. Male PGC enter mitotic arrest at G1/G0 shortly after they reach the gonad and become pro-spermatogonia [10]. In the ovary, retinoic acid is required for the female germ cells to enter meiosis, where they arrest at the first prophase step of meiotic division (germinal vesicle GV). After birth, pro-spermatogonia return to mitotic proliferation forming postnatal spermatogonia stem cells. In the testis of an adult male the functional spermatozoa are produced in each cycle of spermatogenesis, lasting approximately 40 days, and males reach their sexual maturity at 7 months of age, provided, however, that they must be at least 10–12 months old before being used for reproduction [34]. In the case of a female pig, the newborn ovary contains a limited number of oocytes, estimated at 500,000. Female pigs reach sexual maturity at the age of 5–6 months. In the ovary of a sexually mature pig, in each estrous cycle, a given number of primary GV oocytes are recruited and some of them ovulate [35]. It should be noted that besides the gametes (spermatozoa or oocytes), the gonads also contain somatic cells (Sertoli, Leydig) or follicular cells.

### Small non-coding RNA sequencing data

**Figure 1:**
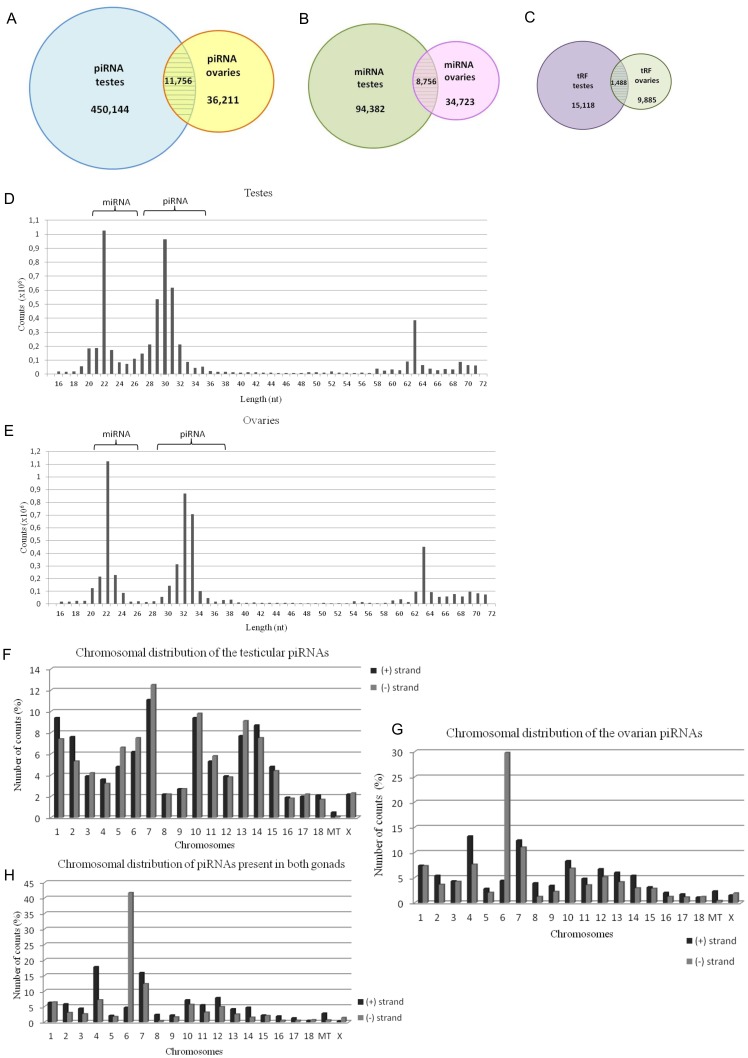
Characteristics of the sncRNA sequences in porcine gonads. SncRNA sequences corresponding to piRNAs (A), miRNAs (B) and tRFs (C) found in the testes and ovaries. In the middle, the size distributions (in nucleotide length) of the sncRNAs isolated from the testes (D) and ovaries (E) are shown. Two separate sncRNA fractions, miRNAs and piRNAs, are marked. At the bottom, the chromosomal distribution of the piRNA sequences is shown: testes-specific (F), ovaries-specific (G), and present in both gonads (H).

To characterize the sncRNAs that accumulate in sexually mature female and male porcine gonads, we extracted total RNA from gonads and included a size selection step to obtain the fractions enriched in sncRNAs. Next, we constructed sncRNA libraries and sequenced them using Illumina technology (Fig. 1A–C). We obtained approximately 6 million reads from each library, of which 4% were low quality and filtered out. Finally 5,393,588 and 5,795,730 reads for the ovaries- and testes-derived sncRNAs, respectively were analyzed. The numbers of unique sequences in the ovaries and testes libraries were 189,119 and 893,561, respectively. The obtained sncRNAs were divided into three groups on base of size and genome mapping; the sncRNAs that were 19–25 nt in length were annotated as miRNA sequences, and the RNAs that were 26–40 nt in length were annotated as piRNA or tRF sequences depend on mapping to tRNA or others non-coding RNA molecules. All sequences were mapped to the genome with 1 mismatch allowed. The library of sncRNAs present in the porcine testes contained 94,382 (11%) unique miRNA sequences, 450,144 (50%) unique piRNA sequences, and 15,118 (2%) unique sequences classified as tRFs. The remaining sequences were shorter than 19 nt or longer than 40 nt (19%) or did not map to the genome (18%). The library of sncRNAs present in the ovaries contained 34,723 (18%) unique miRNA sequences, 46,096 (19%) unique piRNA sequences, and 9885 (5%) tRF sequences. Of the remaining sequences, 40% did not meet the required length criteria, and 18% did not map to the genome. Interestingly, for each sncRNA family, we identified a higher number of unique sequences in the testes than in the ovaries. The ratios of the number of unique small RNA sequences specific to the testes to the number of unique small RNA sequences specific to the ovaries were as follows: ∼12 for piRNAs, ∼3 for miRNAs, and ∼1.5 for tRFs (Fig. 1A–C). In addition, we would like to point out that the number of piRNAs reads determined in our European pig Line 990 model to amount to 450,144 in testes and 46,096 in ovaries, agree with previous studies. In the testes of Meishan pig, 375,195 piRNA reads while 48,620 and 44,309 in GV cumulus and oocyte, respectively, were identified [36],[37].

### piRNAs are present in female and male *S. scrofa* gonads

piRNAs are the most abundant sncRNA sequence type in female and male porcine gonads [23]. Analysis of the piRNA family members present in the ovaries and testes revealed differences in their lengths; the ovarian piRNAs were longer than the testicular piRNAs (Fig. 1D,E). Comparison of the ovary- and testes-derived piRNAs revealed that 11,756 sequences occurred in both gonads (Fig. 1A). Thus, 2.6% of the testes-specific piRNAs and 32.5.% of the ovary-specific piRNAs were present in both male and female gonads.

The piRNA length distribution obtained using the deep-sequencing method demonstrated that the testes-specific piRNA sequences were 29, 30, and 31 nt in length (Fig. 1D). The ovarian piRNAs were 31, 32, and 33 nt in length (Fig. 1E). A similar result was obtained by a standard sequencing analysis of 48 ovaries-specific and 36 testes-specific piRNA sequences [23]. A previous analysis of the piRNA pool isolated from the testes of Chinese Meishan pigs revealed piRNAs that were 1 nt shorter than those observed in much bigger European breed. The most abundant Meishan piRNA sequences were 28, 29, and 30 nt in length [36].

The piRNAs in murine testes range in size from 27 to 30 nt [38]. Less is known about the size of piRNAs in murine ovaries. Standard cloning and sequencing of murine ovarian piRNAs revealed 2 groups with lengths of 27–31 nt and 32–38 nt [39]. However, this analysis was performed on a limited number (dozens) of piRNA sequences. In zebrafish, the testicular and ovarian piRNAs are of similar lengths (26–28 nt) [40]. The above-mentioned sequencing analyses indicate that the size of piRNAs varies depending on the organism. In addition, piRNAs may vary in members of the same species, as shown for different breeds of domestic pigs.

piRNAs form stable ribonucleoprotein complexes with Piwi proteins in the germ cell, including Aub, Piwi and Ago2 in *Drosophila*, Piwil1 (Miwi), Piwil2 (Mili) and Piwil4 (Miwi2) in mouse and pig. However these protein differ in size and sequence [21]–[23]. Thus, the difference in piRNA length could come from the footprint left by the Piwi proteins. Unlike other eukaryotes, in human additional fourth Piwil3 protein was identified [41]. However, applying bioinformatics analysis we did not find open reading frame for this protein in the pig genome.

### Chromosomal distribution of piRNAs

The piRNAs identified in the female and male gonads mapped to the *S. scrofa* genome (Fig. 1F–H). The male piRNAs mapped to all chromosomes but showed a high preference for chromosome 7 (12%) and, to a lesser extent, chromosomes 1, 10, 13, and 14 (8%) (Fig. 1F). In addition, the testicular piRNAs mapped almost equally to the sense and antisense strands of the porcine genome. However, the ovarian piRNAs (30%) mapped with a strong preference to the antisense strand of chromosome 6 (Fig. 1G). A similar preference was observed for the piRNAs detected in both gonads; more than 40% of these piRNA sequences mapped to the antisense strand of chromosome 6 (Fig. 1H). We calculated that up to ten fold fewer piRNA sequences mapped to the sense strand of chromosome 6 than to the antisense strand. Also piRNAs present in pig GV and MII oocytes map prevalently to sense strand of chromosome 6 [37]. In addition, we have calculated that the piRNAs that mapped to chromosomes 4 and 7 were three fold less abundant than the piRNAs that mapped to chromosome 6; however, the latter showed a preference for the sense strand (Fig. 1H). These results also demonstrated that the abundance of piRNA sequences on each chromosome is not related to the locations of the Piwi protein genes. The *S. scrofa piwil1* and *piwil2* genes are encoded on chromosome 14, and *piwil4* is encoded on chromosome 9.

Table S1 shows a fraction of the most abundant piRNAs that are present in both gonads. There are two sets of piRNA molecules: one set contains piRNAs that map to chromosomes once, whereas the other set includes piRNAs that map to chromosomes many times. For example, piRNA no. 39/502 (ovary- and testes-specific identification numbers, respectively) occurs at a frequency of over 18,000 sequences and maps to 17 sites on 5 different chromosomes. However, 69% of the piRNAs mapped to chromosomes only once.

Figures S1A and S1B show the distribution of bases along the lengths of the piRNAs derived from the testes and ovaries. The testicular piRNAs preferentially contained uracil as the first base (over 70%) (Fig. S1A). By contrast, uracil was present as the first base in only 30% of the ovarian piRNAs and the piRNAs present in both gonads (Fig. S1B,C). The ping-pong mechanism proposed for the first time for piRNA biogenesis in *Drosophila* requires the 1U and 10A bias [42]. However, the *S. scrofa* ovary- and testes- specific piRNAs did not possess this characteristic signature, and adenine was present in only 22% of these molecules. The absence of ping-pong signatures is perhaps expected because adult testes and ovaries were examined. Ping-pong signatures like in *Drosophila* were found only in embryonic mouse testes [43],[44]. Therefore, such observation may indicate that these piRNAs are produced via primary processing or other mechanism.

### Clusters of piRNA

**Figure 2:**
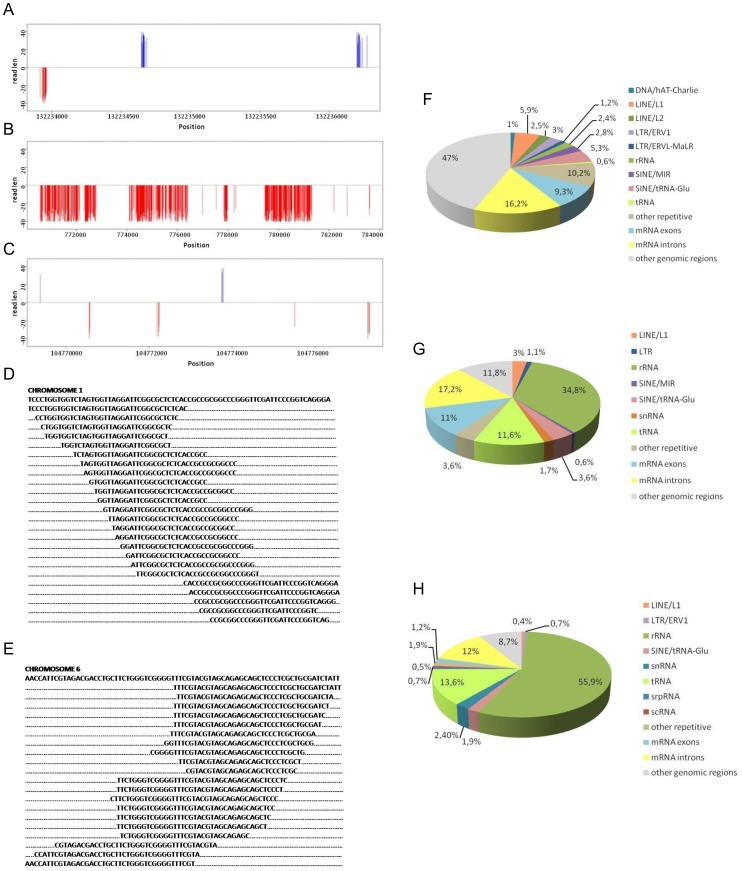
piRNA characterization. The example of clusters of piRNA sequences present in both testes and ovaries on chromosome 1 (A), chromosome 6 (B), and chromosome 4 (C). Next, overlapping piRNA sequences on chromosome 1 (D) and chromosome 6 (E) are shown. On the right, a pie chart summarizing the annotation of the piRNAs matching genomic elements. The following piRNAs are shown: testes-specific (F), ovaries-specific (G), and occurring in both gonads (H).

A distinctive characteristic of piRNAs is their appearance as clusters in a wide range of animal genomes, from fly [42] to mouse [43]. We investigated whether porcine piRNAs present in both gonads also occurred as clusters using the following definition of a cluster: at least 80 piRNAs located not more than 2,500 nt apart on a chromosome. We identified 71 clusters among the shared piRNAs and calculated that a majority of the piRNAs mapped to these discrete loci. Clusters are distributed among all chromosomes, however their number is in range from 1 on chromosome 3 or 15, till 17 on chromosome 6. Previous analysis of Meishan testes specific piRNA allowed to identify 1124 clusters evenly distributed on plus or minus genomic strands [36]. Likewise piRNA derived from *S. scrofa* oocytes and embryos are grouped in clusters, the biggest one was identified on chromosome 6 [37]. Also in *D. melanogaster* and *M. musculus* clusters, piRNAs are derived from one or both genomic strands [42],[44]. The shared *S. scrofa* piRNAs that were present in clusters were observed on either or both genomic strands (Fig. 2A,C, respectively). Thorough analysis of the clusters located on chromosomes 1 and 4 demonstrated that piRNAs were derived from both genomic strands. By contrast, on chromosome 6 of the *S. scrofa* genome, piRNAs were produced from one strand and occurred with a high frequency (Fig. 2B). The high density of this piRNA cluster is consistent with the observations regarding the genomic distributions of the shared piRNAs, i.e., over 40% of the shared piRNA fraction maps to the antisense strand of chromosome 6 (Fig. 1H). These data indicate that piRNA clusters are the source of the piRNAs present in both male and female porcine gonads.

Bioinformatics analysis of the sequencing data showed that a short genomic sequence is the source of many piRNAs because piRNA sequences overlap (Fig. 2D,E). A 71-nt region of porcine chromosome 1 is the source of 24 piRNAs with lengths of 28 to 34 nt. Similarly, a 74-nt region of chromosome 6 is used to generate 20 different piRNAs with lengths of 26 to 33 nt. Overlap of piRNA sequences in cluster regions has also been observed in Meishan pig [36] and in other organisms, such as mouse testes-specific piRNAs [44].

In addition, we mapped *S. scrofa* testicular and ovarian specific piRNAs to the human and mouse genomes. In the case of testes, number of piRNAs which perfectly match (no mismatch) to human and mouse genome is 2.2% and 1.8%, respectively. Such values agree with the recently determined homology of marmoset's piRNAs which map to human and mouse genome in the ratio of 7.3% and 4.5%, respectively [45]. Because most of testicular piRNAs are located in clusters, this analysis confirms that piRNA cluster sequences are not conserved. Interestingly, the homology of ovarian piRNAs to the human and mouse genome is higher and it amounts to 17% for human genome and 14% for mouse genomes. In the case of piRNA fraction present in both gonads these values are higher: as much as 36.1% and 27.1% map to the human and mouse genome, respectively.

### Mapping of piRNAs to RNA species

We mapped the *S. scrofa* piRNAs to RNA species such as retrotransposons, repetitive elements, rRNAs, and tRNAs. The testicular porcine piRNAs mapped to many transposons, including the LINE, SINE, and LTR transposons (Fig. 2F). This observation is consistent with previous reports. In *Drosophila*, both ovary- and testis-specific piRNAs map mainly to transposons [42]. In mice, 17% of the piRNAs that are involved in spermatogenesis map to transposons [43]. Recently, deep sequencing of the testes-specific piRNA pool of Chinese Meishan pigs revealed that the majority of the sequences mapped to transposons [36]. We confirmed these observations and determined that such loci are common for the piRNAs detected in *S. scrofa* testes (Fig. 2F). Notably, 47% of the piRNAs were derived from un-annotated genomic regions, compared with 58% for Meishan pigs [36].

In contrast to the testicular piRNAs, information concerning the genomic distribution of ovarian piRNAs is very limited. Using standard cloning and sequencing methods, 79 mouse ovary-specific piRNAs have been characterized [39]. Approximately 70% of the piRNAs mapped to repetitive regions of the genome. Another analysis using deep sequencing revealed that 21% of piRNAs derived from zebrafish ovaries mapped to repetitive parts of the genome [40].

Our analysis revealed that the annotated piRNAs from porcine ovaries predominantly corresponded (35%) to a particular set of non-coding RNAs, namely, to a ribosomal RNA sequence (Fig. 2G). Furthermore, 12% of the piRNAs mapped to tRNAs, another type of non-coding RNA. The annotated piRNAs common to both male and female gonads mapped almost exclusively to ribosomal RNA and tRNA sequences, 56% and 14%, respectively (Fig. 2H). Therefore, 70% of piRNAs mapped to non-coding RNAs, rRNAs and tRNAs play important roles during protein translation. In eukaryotes, the ribosomal 28S, 18S, 5.8S and 5S RNAs are involved in creating the structure of the ribosome [46]. tRNAs are also critical for translation and are responsible for the proper decoding of genetic information. It is not known whether piRNAs are involved in the regulation of translation. The murine complexes of piRNAs with the Piwil1 (Miwi) protein are associated with polysomes [47]. However, it is difficult to predict whether piRNAs are involved in the regulation of translation.

### Post-transcriptional modifications of gonad-derived piRNAs

**Figure 3:**
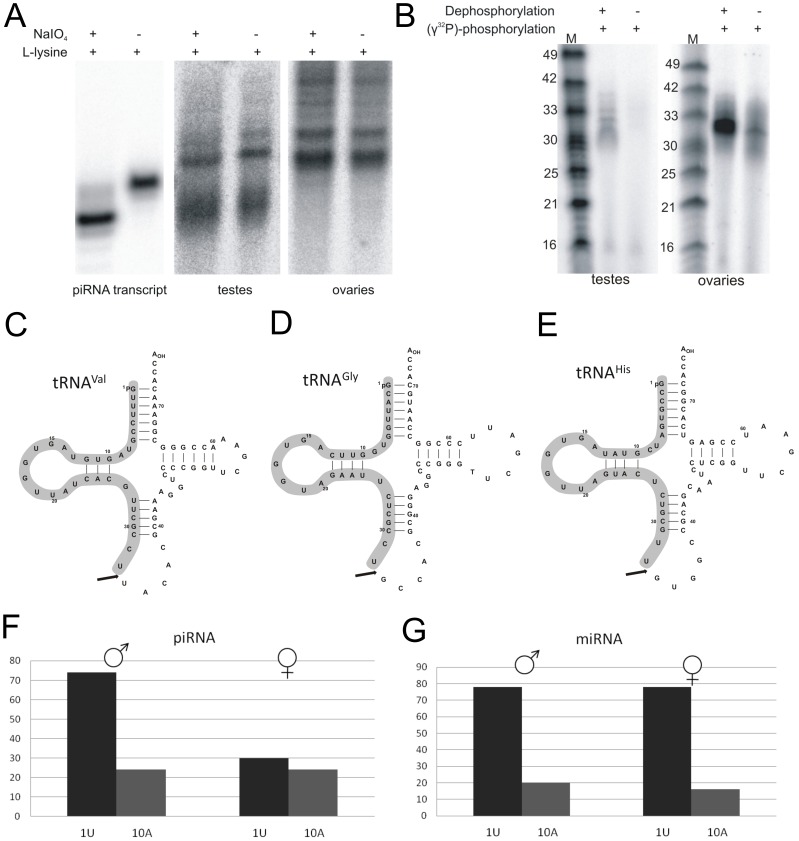
Biogenesis of *S scrofa* piRNAs and tRFs. (Top) An analysis of the post-transcriptional modifications of piRNA at the 3′- (A) and 5′-ends (B) is displayed. To detect 3′-end modifications, the piRNA fraction and the piRNA transcripts were 5′-^32^P end-labeled and treated with NaIO_4_ and L-lysine (A). On the other hand to analyze the 5′-ends of the RNA (B), half of the piRNA pool was dephosphorylated, and both fractions of piRNA were labeled with [γ-^32^P]-ATP using polynucleotide kinase. The RNAs were separated by PAGE. In the middle, the cloverleaf structures of the tRNAs with valine(Val) (C), glycine(Gly) (D), and histidine(His) (E) specificity, which are the main sources of the 5′-tRFs present in both porcine testes and ovaries. The sequences of the tRFs are marked in grey. The arrow indicates the cleavage site in the anticodon loop. (Bottom) The occurrence of uridine and adenosine in the first and tenth positions, respectively, in the nucleotide sequences of the piRNAs (F) and miRNAs (G). The analysis is limited to piRNA and miRNA fraction present in both the male and female gonads.

The piRNAs isolated from different organisms contain modifications such as 2′-O-methylation of the 3′-end of the ribose moiety [48]. These modifications are specific to piRNAs; only plant miRNAs and siRNAs as well as *Drosophila* siRNAs contain 3′-end methylation. We analyzed the methylation patterns in both the female and male piRNA fractions using the piRNA 63/729 transcript as a control (Table S1). This piRNA is present in both gonads, and its transcript was obtained by a standard *in vitro* transcription method that precludes any modification. To probe methylation at the 3′-end of the testicular and ovarian piRNAs, NaIO_4_ oxidation was performed, followed by a β-elimination reaction in the presence of L-lysine (Fig. 3A). When the last nucleotide of the RNA chain contains 2′- and 3′-hydroxyl groups, the β-elimination reaction removes the 3′-nucleotide, generating a shorter RNA molecule. However, the 2′-O-methylated 3′-end of RNA is resistant to the β-elimination reaction. The β-elimination reaction resulted in a mobility difference between treated and untreated piRNA transcript. The piRNAs extracted from gonads were resistant to the probe. Therefore we postulated that the 3′-ends of both the female and male piRNAs contained the 2′-O-CH_3_ modification (Fig. 3A). We also confirmed the presence of a phosphate group at the 5′-end of the piRNAs (Fig. 3B). The piRNA fraction was divided into two parts; one part was subjected to alkaline phosphatase treatment, and both parts were subsequently treated with polynucleotide kinase and (γ-^32^P)-ATP. Only the dephosphorylated piRNA fraction was radiolabeled at a ratio of 40∶1, which indicated the presence of a phosphate group at its 5′-end (Fig. 3B).

The role of this piRNA post-transcriptional modification is unclear, but it might be involved in an interaction of the 5′-phosphate group of piRNA with the MID domain of Piwi proteins [49]. The methyl group at the 3′-end of piRNA enhances the interaction of the PAZ domain of the Piwi proteins with single-stranded piRNA. Furthermore, it has been postulated that the modification of piRNAs increases their stability [50]. In addition, the methylation of animal piRNAs, plant miRNAs, and the *D. melanogaster* Ago2-bound siRNAs protects these sRNAs against the non-templated addition of a 3′-nucleotide [48],[50].

piRNA methylation occurs in a wide range of organisms, from flies to humans. In mouse, the methyl group is introduced at the 3′-end of the RNA chain by the HEN1 methylase [51]. The role of the piRNA-specific HEN1 methylase in germline cells is not well understood; however, deficiency of *Drosophila*-specific methylase, DmHen1, leads to a decrease in the length and abundance of Piwi due to the lack of enzymatic 3′-end methylation of piRNAs [52].

### A majority of the miRNA family present in both porcine gonads is conserved

Consecutive comparative analyses of ovary- and testes-specific miRNAs revealed that 8,756 miRNA sequences were common to the female and male gonads. We then examined the types of genes regulated by these miRNAs (Table S2). We observed high expression of miR-10b, which regulates the activity of the homeobox genes *hox1a* and *hox1b*. These genes are involved in the regulation of embryonic development in a wide spectrum of organisms, from flies to humans [53]. Notably, the abundance of miR10b was similar in female and male gonads (Table S2). Other miRNAs regulate cell proliferation (miR-30b, miR-222, miR-145, and mir-17), aging (miR106a), and central nervous system development (miR184) [54]. Some of these miRNAs are markers for cancers (miR30c-5p, miR-18a, miR-19a, and miR-181b) [55]. Another group of miRNAs regulates metabolic pathways such as the expression of pantothenate kinase. Pantothenate kinase catalyzes the first step of coenzyme A biosynthesis; miRNA-107 controls the level of pantothenate kinase [56]. Furthermore, miRNA-122 controls lipid metabolism [57].

In conclusion, the above analysis indicates a potential role of the identified miRNA family present in both gonads and confirms its importance in the regulation of a wide spectrum of biological processes.

### tRFs in germ cells

Surprisingly, we found that a portion of the analyzed porcine sncRNA fraction mapped to 5′- or 3′- part of tRNA sequences (Table S3 and S4). First group of the porcine 5′-tRFs are mainly derived from the tRNAs with Gly(GCC), His(GTG), and Val(CAC) specificity (Table S3) and are generated by an unknown nuclease that cleaves the tRNA sequence in the anticodon loop (Fig. 3C–E). The second group of 3′-tRFs is derived from 3′ part of tRNAs with Arg(TCT) elongator Met(CAT) and Lys(TTT) specificity and contains a constant CCA sequence from the 3′-end of tRNA (Table S4). Thus, the sources from which the 5′-tRF tRNA and 3′-tRF fragments were obtained are different. We would like to draw attention to the huge difference in the number of 5′ and 3′ tRFs. The highest number of 5′-tRFGly and tRFVal was counted to be almost half million reads each, however, in the case of 3′-tRFArg and tRFMet their number was much smaller, in the range of 1000–4000 reads.

Furthermore, both tRF families are more abundant in the ovaries than in the testes. For example, 5′-tRFGly and tRFVal display a large number of ovary-specific sequences and fewer sequences were observed in the testes (Table S3). The 10 most abundant 5′-tRFs that mapped to the *S. scrofa* genome with no mismatch had 1,244,264 and 50,520 sequences in the ovaries and testes, respectively. Also 3′-tRFs are more abundant in ovaries than in testes (Table S4). Our bioinformatics analysis revealed that these sncRNAs map to the tRNA sequences, which are dispersed among different chromosomes. Also, in other organisms, both 5′- and 3′-tRFs were detected [26]–[28].”

The role of the tRFs in the regulation of gonadal functions is not yet understood. tRFs have been detected in many species, including archaea [58], plants [59],[60], cultured human liver cells [28], and virus-infected human cells [61] Recently, the tRF family was proposed to be involved in stress response and tumor suppression [62]–[64]. The comparison of the so far identified most abundant tRF sequences shows that they differ in size and type of tRNA from which they originated (Table 1). In addition, these observations indicate that tRF can play a variety of functions in the test organisms. In addition some tRFs form complexes with Ago 1 protein and are involved in regulation of gene expression, presumably *via* heterochromatin rearrangement mechanism [65]. Furthermore, the high abundance of 5′-tRFs was detected in mature murine sperm [66]. We demonstrated that such tRFs are also present in porcine gonads. Moreover, the 20-fold higher amount of 5′- and 3′-tRFs in porcine ovaries compared to testes suggests the importance of these sncRNAs in the oogenesis process. This is the first observation of high numbers of tRFs in mammalian ovaries. It will be interesting to determine whether the tRFs form RNA-protein complexes with Piwi proteins. Although the 5′-tRFs form weak complexes with the Argonaute proteins [60], they are unlikely to complex with Piwi proteins because of the low level of expression of the *piwi* genes during porcine oogenesis [23].

**Table 1:** Comparison of the most abundant tRFs identified in different species to present in *Sus scrofa* gonads.

No.	Organism/tissue/cells	tRF type	Length	Reference
**1.**	Archaea (*Haloferax volcanii*)	Val GAC Cys GCA Ser GCT	26 nt 20 nt 20 nt	Gebetsberger et al. [58]
**2.**	*Schistosoma japonicum* eggs	Ala AGC Ala TGC Val TAC Gly GGG	19 nt 19 nt 23 nt 21 nt	Cai et al. [75]
**3.**	*Tetrahymena thermophila*	Asn GTT Gly TCC Gly GCC ThrAGT	30–35 nt 30–35 nt 30–35 nt 30–35 nt	Lee at al. [27]
**4.**	*Arabidopsis thaliana*	Gly TCC Ala AGC Asp GTC	19 nt 16 nt 19 nt	Hsieh et al. [76]
**5.**	Rice (*Oryza sativa*)	Ala AGC Pro CGG	20–22 nt 21 nt	Chen et al. [77]
**6.**	Barley (*Hordeum vulgare*)	His GTG Gly TCC Ala AGC Arg CCT	20 nt 20 nt 20 nt 20 nt	Hackenberg et al. [78]
**7.**	Mouse sperm	Glu TTC Gly GCC Val CAC	31 nt 30 nt 34 nt	Peng et al. [66]
**8.**	Cultured HeLa cells	Gln CTG Val AAC Val CAC Lys TTT	19 nt 19 nt 19 nt 19 nt	Cole et al. [28]
**9.**	Human liver carcinoma cell line (HepG2)	His GTG Ile TAT Glu CTC Asp GTC	37/39 nt 35 nt 22 nt 22/37 nt	Kawaji et al.[79]
**10.**	Sus scrofa	Gly GCC Val TAC His GTG	33 nt 33 nt 33 nt	

### The mechanism of *S. scrofa* piRNA biogenesis

Although miRNA biogenesis pathways have been well described in many reports, piRNA biogenesis in mammals is not yet understood. There is a single working model for the post-transcriptional amplification of piRNAs, which is referred to as the ping-pong model. This model was firstly proposed on the basis of a bioinformatics analysis of piRNAs in *Drosophila* and postulates that long single-stranded RNAs derived mainly from the non-coding parts of the genome (transposons, repetitive sequences, and some introns) are substrates for the biogenesis of piRNAs [42],[43]. An unknown nuclease is responsible for the generation of the primary piRNA set and 5′- and 3′-end formation [67],[68]. Recently, a possible role of the Zucchini nuclease in primary piRNA biogenesis was suggested [69]. Primary piRNAs form RNP complexes with *Drosophila* Piwi and Aub proteins and are in the antisense orientation relative to the transposon sequence, with a strong preference for the 1U bias. These piRNAs are complementary to another group of Ago3-bound piRNAs that contain the 10A bias. Furthermore, the Ago3 and Aub/Piwi-bound piRNAs form base pairs through their first 10 nucleotides [42],[43]. The slicer activity of the Aub protein is involved in the production of secondary piRNAs, which have a 10A bias and are in the sense orientation relative to transposon mRNAs. In the next step of piRNA biogenesis, the secondary sense piRNAs bind to complementary sequences, leading to the production of new antisense piRNAs with a 1U bias. Because the majority of *Drosophila* piRNAs map to defective transposon sequences, the ping-pong mechanism has also been proposed for the transposon silencing process [67].

For murine male piRNA biogenesis like in *Drosophila* two piRNA pathways have been established, primary processing and ping-pong amplification loop [24],[25],[70],[71]. In first pathway genomic loci which encode piRNA are transcribed as long piRNA precursors in size hundreds kb. These RNAs are modified, 5′-capped and 3′-polyadenylated. [70]. Next precursors are processed into shorter piRNA intermediates which associate with Piwil1 (Miwi) and (Piwil2 (Mili) proteins. Such piRNAs show preference to 5′-U bias. In next 3′-end of piRNA intermediates are trimmed into correct 3′-end by some endonuclease [68]. Finally initial pool of mature primary piRNAs is generated. The second pathway utilizes special ping-pong mechanism to generate secondary piRNAs and amplification of both primary and secondary piRNAs. During these mechanism complexes of Piwi proteins with associated primary piRNAs are bound to complementary RNA target and cleave it between 10^th^ and 11^th^ nucleotide producing 5′-end of secondary piRNAs. Thus secondary piRNAs contain special signature the presence of 10A bias. Resulted secondary piRNA are loaded onto Piwi proteins and 3′-end is trimmed. Next during ping-pong mechanism these complexes Piwi proteins with secondary piRNAs are guided to target RNAs, slices them and produce primary piRNAs and amplification loop can be repeated. However, the ping-pong mechanism occur in primordial germ cells of embryonic testes, primary piRNAs are loaded onto Piwil2 (Mili) and secondary piRNAs on both Piwil2 (Mili) and Piwil4 (Miwi2) proteins [70],[71]. Knock out of Piwil2 (Mili) significantly reduces Piwil4 (Miwi2) piRNAs thus pointing to the Slicer activity of the Piwil2 (Mili) protein. Recently, the involvement Piwil4 (Miwi2) piRNAs in *de novo* DNA methylation process which regulates LINE transposon activity in nucleus has been shown [72]. In adult piRNAs associate to Piwil1(Miwi) and Piwil2(Mili) proteins. Yet, the involvement of Piwil1 (Miwi) and Piwil2 (Mili) piRNA in the ping-pong mechanism in adult individuals has recently been questioned [44]. It was shown that the Piwil1 (Miwi)- and Piwil2 (Mili) - associated piRNAs uniquely map to the genome and have no known targets other than their own opposite DNA strand [44].

Furthermore, the *S. scrofa* testes-specific piRNAs have similar characteristics: uracil occurs in the first position of the RNA chain in 70% of the sequences, but only 20% have adenine at the tenth position (Fig. 3F). However, a distinct pattern was observed for the ovary-specific piRNAs (Fig. 3F); similar abundances of uracil and adenine (22–30%) were detected in the 1U and 10A positions of the piRNA sequences. Notably, the porcine miRNAs display features similar to the testes-specific piRNAs because 78% of the miRNA sequences contain the 1U base, and only 18% have the 10A nucleotide (Fig. 3G). Fly, worm, and some mammalian miRNAs contain uracil bias at the 5′-end, which is necessary for the binding of the appropriate Argonaute protein [73]. For example, the mammalian MID domain of the Ago2 protein specifically recognizes miRNAs that begin with a 5′-uracil and the importance of 5′-uracil in the assembly of the RISC complex has been demonstrated [74]. Thus, the 1U bias is not a signature of piRNA biogenesis but is necessary for the loading of Argonaute proteins.

We suggest that the mechanism of *S. scrofa* piRNA biogenesis during spermatogenesis is more similar to that of mouse than to *Drosophila*. First of all, porcine piRNAs are biased at 5′-terminal uracil that occurs in the first position of the RNA chain in 70% of the sequences and with no adenine biased (20%) in the 10^th^ position (Fig. 3F). Such piRNA signature indicates that porcine piRNAs show the characteristics of primary piRNA and the secondary fraction produced by the ping-pong mechanism is at a low level. In addition, the postulated ping-pong mechanism during mouse spermatogenesis requires slicer activity of Piwil2(Mili) and Piwil4(Miwi) proteins [69]. Hovever, in adult pig the expression of porcine *piwil4*, occurring in neonatal male gonad, is 30-fold reduced in adult testes [23]. Taking these remarks together we suggest that the piRNA biogenesis mechanism in adult *S. scrofa* testis is mostly independent of the ping-pong cycle. A similar independent ping-pong mechanism has recently been suggested for piRNA biogenesis in the case of model primate, marmoset [45].

Next are the piRNA biogenesis mechanisms in *S. scrofa* ovaries and testes related? Ovarian piRNAs have different properties, namely, lack of the 1U bias, longer sequences, and different chromosomal and genomic distributions; therefore, their biogenesis might occur by a different mechanism. The slicer activity of Piwi proteins is necessary for piRNA pathway [24],[25]. Moreover, the *piwi* genes are expressed at very low levels in porcine ovaries, at least 10 times lower than in the testes [23]. These observations suggest a different mechanism for piRNA biogenesis during oogenesis. Proteins other than Piwi are potentially involved in this process. However, the details of the alternative mechanism of piRNA biogenesis are unknown.

## Supporting Information

Figure S1 **The base compositions of the piRNA fractions derived from the testes (A), ovaries (B), and piRNAs occurring in both gonads (C).**
(PDF)

Table S1 **The characteristics and abundance of the piRNAs present in** ***S. scrofa*** **female and male gonads.**
(PDF)

Table S2 ***S. scrofa*** **miRNA sequences common to both gonads.** The possible miRNA gene targets and their functions are indicated.
(PDF)

Table S3 **The most abundant 5′-tRF sequences occurring in** ***S. scrofa*** **ovaries and testes.**
(PDF)

Table S4 **The most abundant 3′-tRF sequences occurring in** ***S. scrofa*** **ovaries and testes.**
(PDF)
