# Supplementary figures and images for: Characterization of Sus scrofa Small Non-Coding RNAs Present in Both Female and Male Gonads
Source: PLoS One. 2014 Nov 21;9(11):e113249. doi: 10.1371/journal.pone.0113249 (PMC4240594; doi:10.1371/journal.pone.0113249)

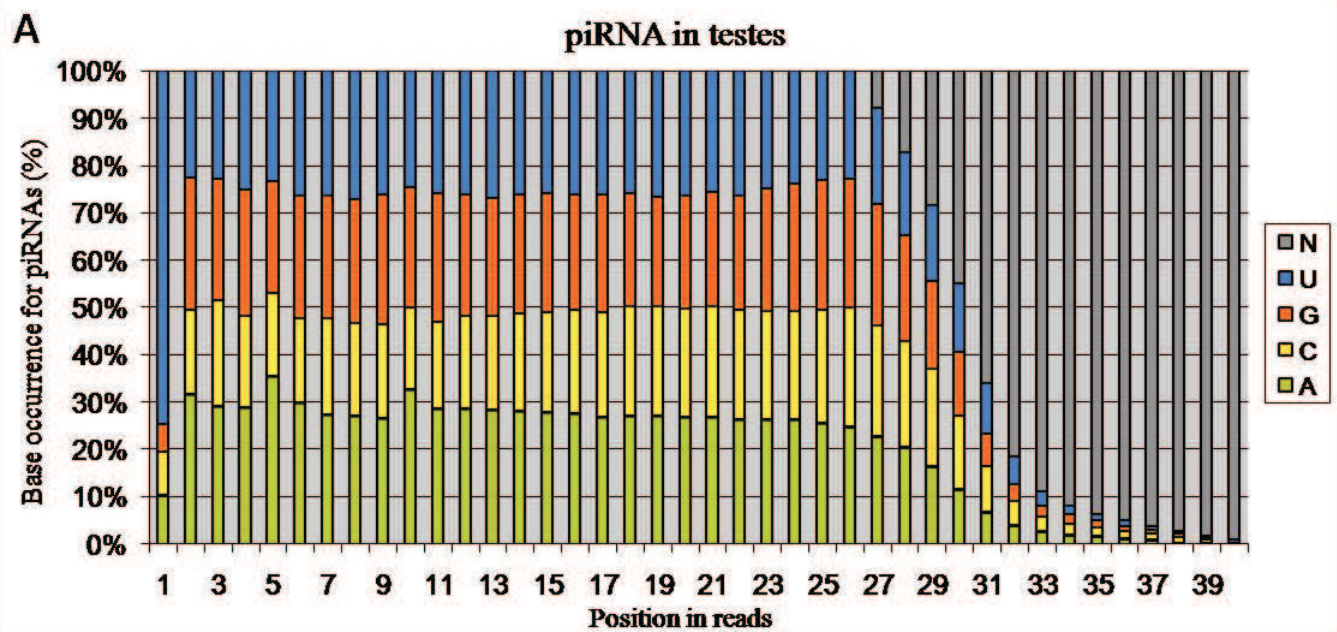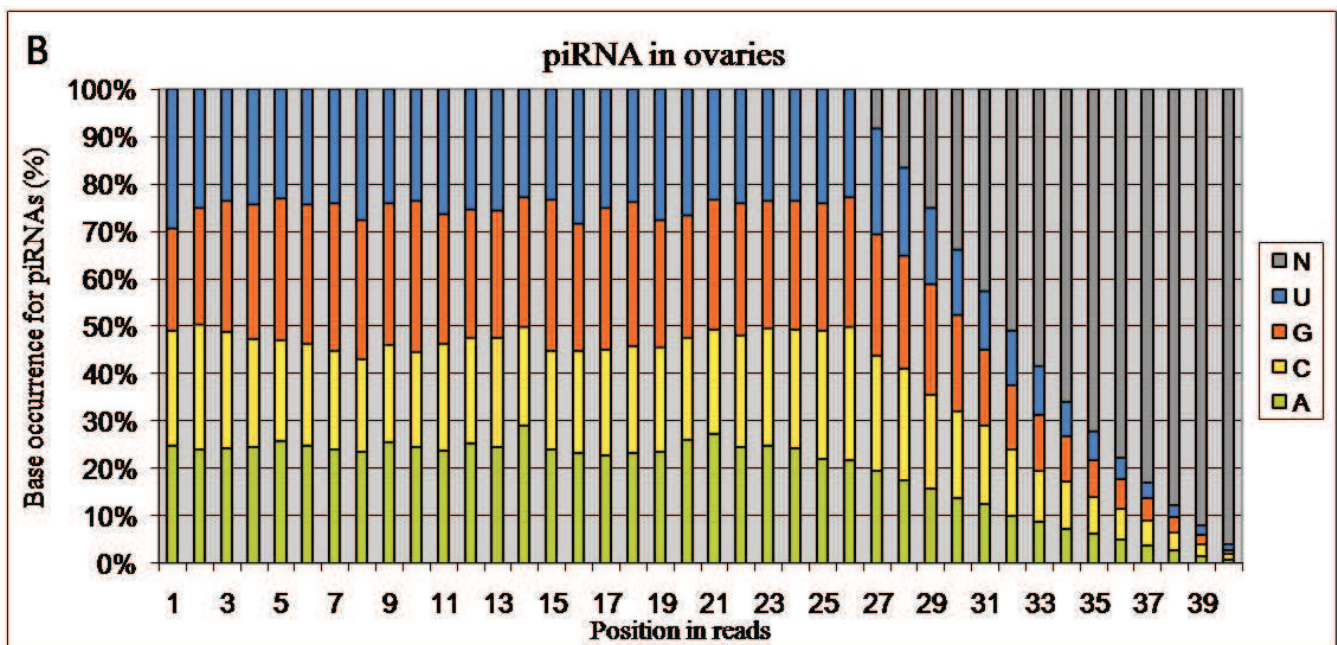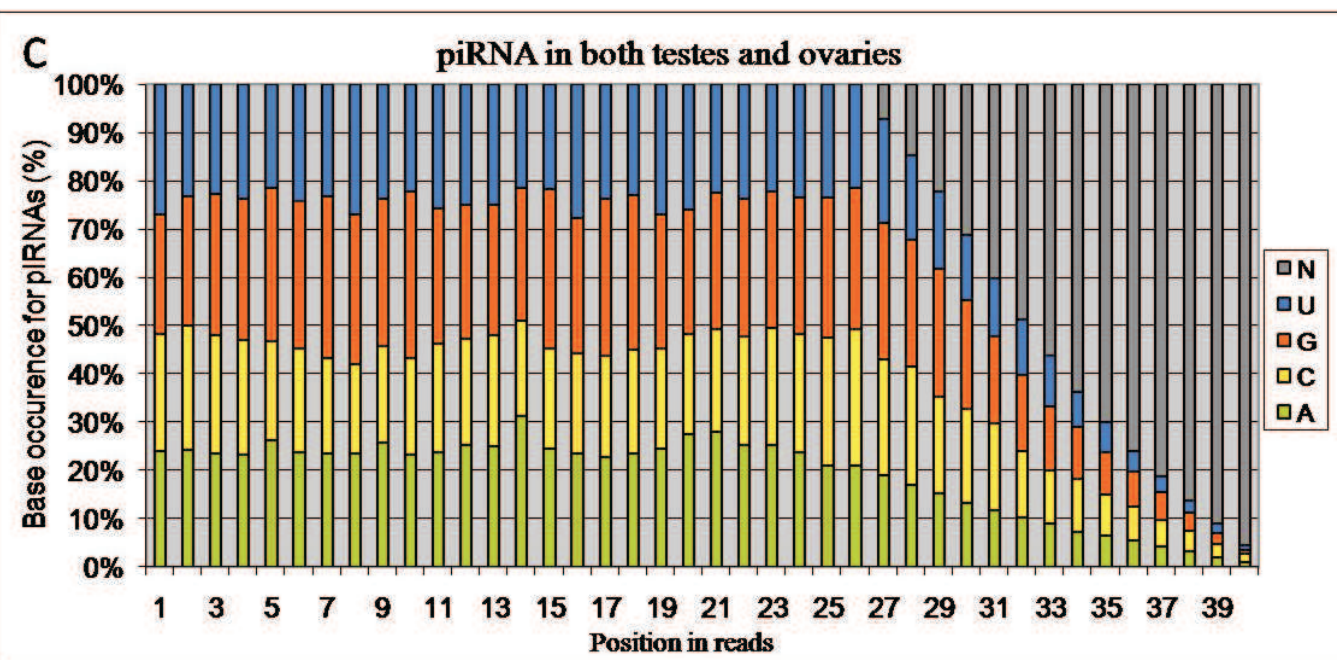

Supplement: Figure S1 — The base compositions of the piRNA fractions derived from the testes (A), ovaries (B), and piRNAs occurring in both gonads (C). (PDF) [file pone.0113249.s001.pdf]
