# Supplementary material for: Characterization of Sus scrofa Small Non-Coding RNAs Present in Both Female and Male Gonads
Source: PLoS One. 2014 Nov 21;9(11):e113249. doi: 10.1371/journal.pone.0113249 (PMC4240594; doi:10.1371/journal.pone.0113249)
Supplement: Table S1 — The characteristics and abundance of the piRNAs present in S. scrofa female and male gonads. (PDF) [file pone.0113249.s002.pdf]

Table 1. The characteristics and abundance of the piRNAs present in *S.srofa* female and male gonads.

	Number of sequences		Number of sequences		Number of matches			First match	
Sequence id ovaries		Sequence id testes		Sequence		Size (nt)	Strand	Chromo-some	Position
6	116545	92	4932	GCATTGTGGTTCAGTGGTAGAATTCTCGCCT	1	31	-	10	58782524
8	84959	339	1110	GCATGGGTGGTTCAGTGGTAGAATTCTCGCCT	2	32	-	1	276742124
17	39355	246	1475	GCATGTGGTTCAGTGGTAGAATTCTCGCCT	2	30	+	4	20495382
18	35080	572	692	GGCCGTGATCGTATAGTGGTTAGTACTCTGCGTT	9	34	+	1	132234645
7485	13	13	29335	TAAGAACTGAGAGATTGTGAACCTTGGCCC	1	30	+	1	261405993
25	25619	275	1328	GCATTGTGGTTCAGTGGTAGAATTCTCGCC	1	30	-	10	58782525
15683	6	17	22761	TCAATGAATAAGAGCAACGTTCTGGCACGT	2	30	+	14	87378312
34	18315	693	582	GCATTGGTGGTTCAGTGGTAGAATTCTCGCCA	14	32	+	12	55953777
38	17642	1058	380	GCCCGGCTAGCTCAGTCGGTAGAGCATGGGACT	2	33	+	7	53403061
39	17223	502	774	GTTTCCGTAGTGTAGTGGTTATCACTTTCGCCT	17	33	+	13	114880302
41	16332	681	594	GTTTCCGTAGTGTAGTGGTTATCACGTTCGCCA	17	33	+	13	114880302
11917	8	23	16327	TTTAAGGTGTTTGTTGCAGCTTTTGTAGCCT	1	31	+	2	18514728
44	15028	412	915	GTTTCCGTAGTGTAGTGGTTATCACGTTCGCCTC	12	34	+	14	107482701
48	14261	625	643	GCATTTGTGGTTCAGTGGTAGAATTCTCGCCT	14	32	+	12	55953777
84	7618	77	6199	GTACATGATGACAACTGGCTCCCTCTAC	1	28	-	1	33085373
28421	3	30	11786	TCTTTCCAAAGCAAAATATCATAGGGACTT	1	30	+	7	90168570
14421	6	32	11598	TAGGCAAACGTGACATGTCTGTTTCGGACC	1	30	-	5	7631172
82	7677	122	3569	GGCTGGTCCGATGGTAGTGGGTCATCAGAACT	1	32	+	10	6079078
63	10578	729	553	GCATGTGGTTCAGTGGTAGAATTCTCGCC	2	29	+	4	20495382
18400	5	40	10438	TAATTCGTCGTGTGAATCTGTCACTTGGC	1	29	-	7	34592571
26894	3	41	10257	TAAATCACTGTGGAAAAGTAGCTCTCCATT	1	30	+	1	125456109
95675	1	42	9706	TAGACTGTTTTTAGGATATCAGATTCTGCCT	1	31	-	7	115837458
44145	2	43	9603	TCGGCAATGACCATACAAGGAAGGTAGGAGC	1	31	-	6	39387801
182068	1	44	9586	TAGACATCTGAGAACAAGTAAACCTGAACCT	1	31	-	7	91143665

93	6225	139	2967	TGTACATGATGACAACTGGCTCCCTCTAC	1	29	-	1	33085373
23624	3	46	9088	TATTAATAGTAGAAGGCCTTTAACCCAGAA	1	30	+	7	90167971
76	8669	1226	326	GCATTGGTGGTTCAATGGTAGAATTCTCGCCT	1	32	+	4	104735772
19599	4	49	8940	TAGAAAACATGAATGGCACCCAAACTATAGG	1	31	+	14	53951797
179692	1	51	8825	TTATCCGTAAATGTGGAGCTGAACTAGCCCA	1	31	-	14	78534128
92	6379	222	1661	AAGCTATGATGATTTCGATTGCATTGATCATA	2	32	-	X	118532802
30608	3	60	8020	TTCCAAGGAAAATCACAATTCTGAATTACC	1	30	-	5	7631315
159528	1	64	7674	TCAATAAATATTTGTAGAATGCATGAAGGG	1	30	+	7	34609003
49186	2	66	7466	TAAGAACTGAGAGATTGTGAACCTTGGCC	1	29	+	1	261405993
103	5666	215	1702	GCATTGGTGGTTCAGTGGTAGAATTCTCGCCTGC	10	34	+	12	55953777
41518	2	67	7118	TTTGTTAGAAATGCAAATTCTGGGGCCC	1	28	-	13	131337158
98471	1	68	6975	TTCACTGACAGCTTCTGATGTGCTGGGAGG	1	30	+	7	91180100
41838	2	69	6796	TGGACTGGGAATCAGACTTGGAGCGCAATC	1	30	-	13	77036140
101	5702	390	967	AGTTGGTCCGAAGGCTGTGGGTTATTGTTAACT	2	33	+	13	173935499
45286	2	72	6662	TAACATTTGGCTGTATGAACTTCTGCTAAA	1	30	-	7	91151598
46065	2	73	6602	TAACGTACGGACCATATGGAACTCACCATC	1	30	+	7	34616918
30562	3	75	6443	TAAACTGTACTGAGCGATTCCAGAAATAGC	1	30	+	14	87376022
175	2771	126	3461	GCATTGGTGGTTCAGTGGTAGAATTCTCGCCTGCC	10	35	+	12	55953777
18983	4	78	6160	TGAAGCATCGATTTGAATTCCAAGGACACTG	1	31	-	5	7631278
99	5801	4243	91	GTTTCCGTAGTGTAGTGGTTATCACGCTCGCCT	2	33	-	7	22458059
102	5667	1825	213	GCATTGGTCGTTCAGTGGTAGAATTCTCGCCT	14	32	+	12	55953777
159694	1	80	5770	TTGGATAGACATCTGAGAACAAGTAAACCT	1	30	-	7	91143671
16580	5	81	5757	TCTCTAGGTAATTAGCTGACACTGGCACCC	1	30	+	7	87266268
106	5389	1101	366	AGCAGAGTGGCGCAGCGGAAGCGTGCTGGGC	11	31	+	3	28905481
105	5596	3253	118	GCATGGGTGGTTCAGTGGTAGAATTCTCGCC	2	31	-	1	276742125
1052	199	87	5422	TCCCACATGGTCTAGCGGTTAGGATTCCTG	3	30	+	11	22178191
21366	4	85	5563	TAGTTGACCTTGAGACACCTTGACTTGGGCC	1	31	+	14	53955622
114	4938	2307	167	TCCCTGGTGGTCTAGTGGTTAGGATTCGGCGC	9	32	+	12	7907937
37981	2	90	5011	TAAACACATTACTGAAATCAGAGGACAATG	1	30	+	14	53948333
33582	2	91	4967	TTGGACTTTTAATCTGTTGGCAATGGGAA	1	29	-	7	91146589
123	4444	790	516	AGTTGGTCCGAAGGCTGTGGGTTATTGTTAAC	2	32	+	13	173935499
122	4487	1167	346	GCCCGGCTAGCTCAGTCGGTAGAGCATGAGACTC	8	34	+	3	39947608
232	1752	136	3038	GGCCGTGATCGTATAGTGGTTAGTACTCTGC	9	31	+	1	132234645

125	4348	1186	340	GCCGTGATCGTATAGTGGTTAGTACTCTG	10	29	+	1	132234646
171286	1	98	4623	GAGACAGACTTTTAACTTTTTGAGCAAAGT	1	30	+	14	53948109
192	2383	174	2183	GGCTGGTCCGATGGTAGTGGGTCATCAGAAC	1	31	+	10	6079078
45242	2	101	4516	TACAATCTGGTAAGGGACACAAGACAGCACT	1	31	-	7	34588989
124	4356	3230	119	TGCCGTGATCGTATAGTGGTTAGTACTCTGCGTT	1	34	+	5	56138058
134389	1	105	4297	TGGGAATTCGCCTCTGAACAAGCCAGACAGG	1	31	+	7	34625902
129	3922	2510	154	GTTTCCGTAGTGTAGTGGTTATCACATTCGCCT	18	33	+	13	114880302
248	1523	158	2479	GGCCGTGATCGTATAGTGGTTAGTACTCTG	10	30	+	1	132234645
155121	1	115	3732	TACTGTGAGAATTTTTGTGCCAATTGGTAGA	1	31	+	1	125447803
165	2941	532	742	GCATTGGTGGTTCAGTGGTAGAATTCTCGCCTG	10	33	+	12	55953777
121364	1	118	3660	TCAATAAATATTTGTAGAATGCATGAAGG	1	29	+	7	34609003
38411	2	120	3581	TGAATTAGGCACTGTGATCTCGTCCAGAAG	1	30	+	6	90947560
170617	1	121	3575	TAATGAGTGTAGCGAGTAGGACCCGGAGA	1	29	-	5	67574847
33501	2	124	3536	ATGACTGTGAGGCACAGAAACAACATGGCACC	1	32	+	7	34610657
71695	1	125	3484	TAGTTTTAGAAGACAAAGAGAACCTGGCATC	1	31	-	3	8078962
144	3311	2975	129	GGGGGTATAGCTCAGTGGTAGAGCATTTGACT	11	32	+	12	24465200
16610	5	129	3397	TACGAGATTGGTAAGAATTAAAAGGTTATT	1	30	-	5	7619752
43903	2	130	3380	TTTGAAATCAGGACTGTTCTGGAAAGTCC	1	29	-	7	115833819
149	3245	3191	120	GCATTGGTAGTTCAGTGGTAGAATTCTCGCCT	14	32	+	12	55953777
152	3191	3015	128	GTTTCCGTAGTGTAGTGGTTATCACCTTCGCCT	17	33	+	13	114880302
249	1522	207	1790	TTCCATGAGTGAAATCTAATAGTCTGACA	1	29	-	1	33086430
167	2900	1518	259	TCCCATATGGTCTAGCGGTTAGGATTCCTGGTT	1	33	+	15	34740883
333	1011	182	2122	TGTCTATGATGATCCTATCCCGAACC	2	26	+	1	58110462
379	840	176	2159	TGGGGGGCCCAAGTCCTTCTGATCGAGGCCC	2	31	-	18	20869690
42146	2	140	2960	TGGTCAAAGAATCATATCTGTAAAATAGAAT	1	31	+	7	90168010
176	2752	2441	158	GCATTGGTGGTTCAATGGTAGAATTCTCGCC	1	31	+	4	104735772
147986	1	143	2866	TTGGAGAATTCTGGACCATGGCTCTGGGT	1	29	+	7	34604658
393	782	195	1964	GCCCGGCTAGCTCAGTCGGTAGAGCATGG	2	29	+	7	53403061
48269	2	148	2699	TTTGTTAGAAATGCAAATTCTGGGGCC	1	27	-	13	131337159
186	2513	3411	112	GCATTTGTGGTTCAGTGGTAGAATTCTCGCC	14	31	+	12	55953777
190	2475	5107	75	GCCCGGCTAGCTCAGTCGGTAGAGCATGGGAC	2	32	+	7	53403061

Abundance of testicular piRNAs is marked by blue, ovarian by red
