# Supplementary material for: Characterization of Sus scrofa Small Non-Coding RNAs Present in Both Female and Male Gonads
Source: PLoS One. 2014 Nov 21;9(11):e113249. doi: 10.1371/journal.pone.0113249 (PMC4240594; doi:10.1371/journal.pone.0113249)
Supplement: Table S2 — S. scrofa miRNA sequences common to both gonads. The possible miRNA gene targets and their functions are indicated. (PDF) [file pone.0113249.s003.pdf]

Table S2. *S. scrofa* miRNA sequences common to both gonads. The possible miRNA gene targets and their functions are indicated.

Id of sequences second (blue) and four (red) numbers indicate abun-dance in ovaries and testes, respectively	Size (nt)	Sequence	Id_of gene to which miRNA sequences are mapping	Id miRNA species from mirBase	Possible miRNA function in the cell	Match quality
19-32075-25-14976	23	TACCCTGTAGAACCGAATTTGTG	NR_035383.1	miR-10b	Regulation homebox*HOXA1a* and *HOXA3a* genes	6E-9
28-22700-11-32181	21	ACCCTGTAGAACCGAATTTGT	NR_035383.1	miR10b	Regulation homebox*HOXA1a* and *HOXA3a* genes	8E-8
47-14306-24-16242	23	ACCCTGTAGAACCGAATTTGTGT	NR_035383.1	miR 10b	Regulation homebox*HOXA1a* and *HOXA3a* genes	4E-9
50-14010-57-8542	23	ACCCTGTAGAACCGAATTTGTG	NR_035383.1	miR 10b	Regulation homebox*HOXA1a* and *HOXA3a* genes	5E-9
334-1007-348-1073	20	TGTAAACATCCCCGACTGGA	NR_035380.1	miR-30b	Regulation integrin protein	4E-10
372-855-3907-298	22	AAAAGCTGGGTTGAGAGGGCGA	NR_035382.1	miR320	Regulation of transferin receptor	3E-10
374-854-1280-314	23	AACATTCATTGCTGTCGGTGGGT	NR_031534.1	miR-181b	Regulation NR6A1 nuclear receptor, cancer marker	2E-09
480-593-773-526	22	TGTAAACATCCTACACTCAGCT	NR_035368.1	30c-5p	Regulation a number of breast cancers	2E-10
507-556-696-580	22	AGCAGCATTGTACAGGGCTATC	NR_031539.1	miR-107	PANK1 pantothenate kinase 1	2E-07
529-526-1326-302	22	AACATTCATTGCTGTCGGTGGG	NR_031534.1	miR-181b	Regulate NR6A1 nuclear receptor, cancer marker	7E-10
586-462-3944-97	24	AACATTCATTGCTGTCGGTGGGTT	NR_031534.1	miR-181b	Regulate NR6A1 nuclear receptor, cancer marker	7E-10
612-439-1045-383	21	AGCAGCATTGTACAGGGCTAT	NR_031539.1	miR-107	PANK1 pantothenate kinase 1	6E-11
731-343-466-826	24	TGAGAACTGAATTCCATAGGCTGT	NR_035377.1	miR-146	Regulation of inflamation	3E-9
5,00E-9	21	TTCCTATGCATATACTTCTTT	NR_035399.1	mirR-465a-3p	Regulation germ cell transition protein (Tnp2), found in mouse oocyte	5E-8
832-280-924-437	23	AGCTACATTGTCTGCTGGGTTTC	NR_035371.1	miR-221	Regulation of angiogenesis,	3E-8

835-279-5430-70	21	TGTAAACATCCCCGACTGGAA	NR_035380.1	miR-200	The miR-200 family inhibits the initiating step of metastasis, epithelial-mesenchymal transition	2E-10
866-259-1288-312	24	TCAGTGCATCACAGAACTTTGTTT	NR_035387.1	miR-148a-3p	Regulation proliferation and growth of t cell, cancer marker	7E-9
885-253-840-484	22	AGCTACATTGTCTGCTGGGTTT	NR_035371.1	miR 221	Regulation of angiogenesis,	5E-8
1119-183-6665-356	23	CAAAGTGCTTACAGTGCAGGTAG	NR_031541.1	miR17	In mice deletion of miR-17 is lethal and causes lung and lymphoid cell developmental defects.	4E-8
1235-157-670-601	24	AGCTACATCTGGCTACTGGGTCTC	NR_035397.1	miR-222	Regulation of human embryonic stem cells expression	9E-8
1260-153-2126-184	23	TCAGTGCATCACAGAACTTTGTT	NR_035387.1	miR-148a-3p	Regulation proliferation and growth of t cell, cancer marker	7E-9
1119-183-6665-56	23	CAAAGTGCTTACAGTGCAGGTAG	NR_031541.1	miR-106a	Down-regulated in human aging	2E-9
1173-170-19600-18	23	TCCCTGTCCTCCAGGAGCTCACT	XM_00312425 3.2	miR-28-3p	Uncharacterized protein C7orf50-like	8E-10
1183-168-5514-69	23	AACATTCATTGCTGTCGGTGGGA	NR_031534.1	miR-181b	Regulate NR6A1 nuclear receptor, cancer marker	2E-04
1213-161-1200-335	22	TCAGTGCATCACAGAACTTTGT	NR_035387.1	miR-148a-3p	Regulation proliferation and growth of t cell, cancer marker	8E-10
2835-97-5996-63	22	AACATTCATTGCTGTCGGTGGA	NR_031534.1	miR-181b	Regulation NR6A1 nuclear receptor, cancer marker	4E-5
2044-74-6174-61	24	CAAAGTGCTTACAGTGCAGGTAGT	NR_031541.1	miR-106a	Down-regulated in human aging	3E-9
2684-50-16453-21	21	AGGGTTGGGCGGAGGCTTTCC	NR_037200.1	miR-411a	Imprinted miRNA suppressing myogenic factor	8E-10
3094-41-33227-10	21	TGTGCAAATCTATGCAAAACTG	XM_00312425 3.2	miR-28-3p	Regulation proliferation and growth of the cell, cancer marker	7E-10
2424-58-25881-13	22	TGGACGGAGAACTGATAAGGGT	NR_031535.1.	miR-184	Regulation the development of embryo, especially the central nervous system	7E-7
3663-32-9494-39	21	TGTAAACATCCTACACTCAGC	NR_035368.1	miR-30c-5p	Regulation a number of breast cancer	7E-10
3787-31-18570-19	23	TGTGCAAATCTATGCAAAACTGA	NR_031537.1	miR-19a	This miRNA belongs to a polycistronic miRNA cluster that is often overexpressed in solid cancers.	2E-7
