# Supplementary material for: Characterization of Sus scrofa Small Non-Coding RNAs Present in Both Female and Male Gonads
Source: PLoS One. 2014 Nov 21;9(11):e113249. doi: 10.1371/journal.pone.0113249 (PMC4240594; doi:10.1371/journal.pone.0113249)
Supplement: Table S3 — The most abundant 5′-tRF sequences occurring in S. scrofa ovaries and testes. (PDF) [file pone.0113249.s004.pdf]

Table S3. The most abundant 5' tRF sequences present in *S. scrofa* ovaries and testes.

Size (nt)	tRF sequence	Sequence Id and abundance of tRFs in ovaries and testes				Match to corresponding tRNA sequence	Match quality
32	GCATTGGTGGTTCAGTGGTAGAATTCTCGCCT	1	598847	14	24085	chr7.trna1931-GlyGCC	6E-14
33	GTTTCCGTAGTGTAGTGGTTATCACGTTCGCCT	2	493802	20	21124	chr7.trna173-ValCAC	2E-14
31	GCATTGGTGGTTCAGTGGTAGAATTCTCGCC	7	98576	109	4023	chr7.trna1931-GlyGCC	2E-13
33	GCCGTGATCGTATAGTGGTTAGTACTCTGCGTT	21	30879	936	431	chr4.trna639-HisGTG	2E-14
32	GTTTCCGTAGTGTAGTGGTTATCACGTTCGCC	35	18260	788	516	chr7.trna173-ValCAC	6E-14
33	GCCCGGCTAGCTCAGTCGGTAGAGCATGAGACT	51	13998	1480	266	chr3.trna319-LysCTT	2E-14
33	TCCCTGGTGGTCTAGTGGTTAGGATTCGGCGCT	60	11045	606	660	chr12.trna51-GluTTC	2E-14
30	GCCGTGATCGTATAGTGGTTAGTACTCTGC	69	9683	249	1451	chr4.trna639-HisGTG	8E-13
30	GCATTGGTGGTTCAGTGGTAGAATTCTCGC	73	9216	379	996	chr7.trna1931-GlyGCC	8E-13
32	GCATTGGTGGTTCAATGGTAGAATTCTCGCCT	76	8669	1226	326	chr4.trna640-GlyCCC	6E-14
34	GCATTGGTGGTTCAGTGGTAGAATTCTCGCCTGC	103	5666	215	1702	chr7.trna1931-GlyGCC	4E-15
35	GCATTGGTGGTTCAGTGGTAGAATTCTCGCCTGCC	175	2771	126	3461	chr7.trna1931-GlyGCC	1E-15
33	GTTTCCGTAGTGTAGTGGTTATCACGCTCGCCT	99	5801	4243	91	chr7.trna1918-ValAAC	2E-14
31	AGCAGAGTGGCGCAGCGGAAGCGTGCTGGGC	106	5389	1101	366	chr7.trna1932-MetCAT	2E-13
30	TCCCACATGGTCTAGCGGTTAGGATTCCTG	1052	199	87	5422	chr1.trna934-GluTTC	8E-13
32	TCCCTGGTGGTCTAGTGGTTAGGATTCGGCGC	114	4938	2307	167	chr12.trna51-GluTTC	6E-14
34	GCCCGGCTAGCTCAGTCGGTAGAGCATGAGACTC	122	4487	1167	346	chr3.trna319-LysCTT	4E-15
29	GCCGTGATCGTATAGTGGTTAGTACTCTG	125	4348	1186	340	chr4.trna639-HisGTG	3E-12
33	GCATTGGTGGTTCAGTGGTAGAATTCTCGCCTG	165	2941	532	742	chr7.trna1931-GlyGCC	2E-14
33	TCCTCGTTAGTATAGTGGTGAGTATCCCCGCCT	162	3083	14008	25	chr7.trna198-AspGTC	2E-14
36	GCATTGGTGGTTCAGTGGTAGAATTCTCGCCTGCCA	648	408	160	2439	chr7.trna1931-GlyGCC	3E-16
29	GCCCGGCTAGCTCAGTCGGTAGAGCATGG	393	782	195	1964	chr7.trna506-LysCTT	3E-12
32	GCCCGGCTAGCTCAGTCGGTAGAGCATGGGAC	190	2475	5107	75	chr7.trna506-LysCTT	6E-14
32	GCCCGGCTAGCTCAGTCGGTAGAGCATGAGAC	187	2496	7047	53	chr3.trna319-LysCTT	6E-14

33	TCCCACATGGTCTAGCGGTTAGGATTCCTGGTT	212	2055	2991	129	chr1.trna934-GluTTC	2E-14
31	GCCGTGATCGTATAGTGGTTAGTACTCTGCG	219	1934	5190	74	chr4.trna639-HisGTG	2E-13
30	TCCCTGGTGGTCTAGTGGTTAGGATTCGGC	768	315	229	1602	chr12.trna51-GluTTC	8E-13
26	GAACAATAGGACTCGAACCTAAACCT	608	445	294	1249	chrM.trna3-GlnTTG	2E-10
34	TCCCTGGTGGTCTAGTGGTTAGGATTCGGCGCTC	282	1282	1217	329	chr7.trna249-GluCTC	4E-15
33	GCCCGGATAGCTCAGTCGGTAGAGCATCAGACT	261	1457	10982	33	chr7.trna248-LysTTT	2E-14
31	GAACAATAGGACTCGAACCTAAACCTGAGAA	422	719	548	722	chrM.trna3-GlnTTG	2E-13
32	GCCCGGATAGCTCAGTCGGTAGAGCATCAGAC	276	1332	9355	39	chr7.trna248-LysTTT	6E-14
30	ATTGGTGGTTCAGTGGTAGAATTCTCGCCT	288	1237	4231	91	chr7.trna1931-GlyGCC	8E-13
35	GCCCGGCTAGCTCAGTCGGTAGAGCATGGGACTCT	330	1020	1424	277	chr7.trna506-LysCTT	1E-15
33	TCCTCGTTAGTATAGTGGTTAGTATCCCCGCCT	283	1280	33891	10	chr5.trna839-AspGTC	2E-14
32	AGCAGAGTGGCGCAGCGGAAGCGTGCTGGGCC	286	1240	12670	28	chr7.trna1932-MetCAT	6E-14
33	AGCAGAGTGGCGCAGCGGAAGCGTGCTGGGCCC	319	1071	2226	174	chr7.trna1932-MetCAT	2E-14
28	GCCGTGATCGTATAGTGGTTAGTACTCT	346	975	1893	206	chr4.trna639-HisGTG	1E-11
35	TCCCTGGTGGTCTAGTGGTTAGGATTCGGCGCTCT	327	1028	2544	152	chr7.trna249-GluCTC	1E-15
33	GAACAATAGGACTCGAACCTAAACCTGAGAATT	491	579	717	565	chrM.trna3-GlnTTG	2E-14
34	GGTTCCATGGTGTAATGGTTAGCACTCTGGACTC	322	1056	39645	8	chr7.trna199-GlnCTG	4E-15
30	GAACAATAGGACTCGAACCTAAACCTGAGA	444	668	1021	393	chrM.trna3-GlnTTG	8E-13
37	GAACAATAGGACTCGAACCTAAACCTGAGAATTCAAA	468	613	1115	362	chrM.trna3-GlnTTG	8E-17
32	GAACAATAGGACTCGAACCTAAACCTGAGAAT	555	499	884	456	chrM.trna3-GlnTTG	6E-14
38	GAACAATAGGACTCGAACCTAAACCTGAGAATTCAAAA	439	685	1492	264	chrM.trna3-GlnTTG	2E-17
32	GCCGTGATCGTATAGTGGTTAGTACTCTGCGT	366	879	16362	22	chr4.trna639-HisGTG	6E-14
32	GGCTCGTTGGTCTAGGGGTATGATTCTCGCTT	387	814	38984	8	chr3.trna1430-ProTGG	6E-14
27	GCCGTGATCGTATAGTGGTTAGTACTC	421	721	4438	87	chr4.trna639-HisGTG	4E-11
30	TCCCATATGGTCTAGCGGTTAGGATTCCTG	1862	85	571	693	chr15.trna1331-GluTTC	8E-13
30	GCCCGGCTAGCTCAGTCGGTAGAGCATGGG	1161	172	690	586	chr7.trna506-LysCTT	8E-13
34	GGGGGTATAGCTCAGTGGTAGAGCATTTGACTGC	568	484	1502	262	chr12.trna209-CysGCA	4E-15
33	GGTTCCATGGTGTAATGGTTAGCACTCTGGACT	407	733	63380	5	chr7.trna199-GlnCTG	2E-14
34	GCCGTGATCGTATAGTGGTTAGTACTCTGCGTTG	428	708	15556	23	chr4.trna639-HisGTG	4E-15
31	TTCCGTAGTGTAGTGGTTATCACGTTCGCCT	682	376	1415	279	chr7.trna173-ValCAC	2E-13
26	GCCCGGCTAGCTCAGTCGGTAGAGCA	548	503	2674	145	chr3.trna319-LysCTT	2E-10

Abundance of testicular tRFs is marked by blue, ovarian by red
