# Supplementary material for: Characterization of Sus scrofa Small Non-Coding RNAs Present in Both Female and Male Gonads
Source: PLoS One. 2014 Nov 21;9(11):e113249. doi: 10.1371/journal.pone.0113249 (PMC4240594; doi:10.1371/journal.pone.0113249)
Supplement: Table S4 — The most abundant 3′-tRF sequences occurring in S. scrofa ovaries and testes. (PDF) [file pone.0113249.s005.pdf]

Table S4. The most abundant 3' tRF sequences occurring in *S. scrofa* ovaries and testes.

Size (nt)	tRF sequence	Sequence Id and abundance of tRFs in ovaries and testes				Match to corresponding tRNA sequence	Match quality
38	TTCAAAGGTTCCGGGTTCGGGTCCCGGCGGAGTCGCCA	133	3717	12709	28	chr4.trna909-ArgTCT	3E-13
37	TCAAAGGTTCCGGGTTCGTGTCCCGGCGGAGTCGCCA	151	3196	11853	31	chr4.trna909-ArgTCT	1E-12
36	TCAAAGGTTCCGGGTTCGGGTCCCGGCGGAGTCCCA	160	3086	16043	22	chr4.trna909-ArgTCT	1E-12
38	TTCAAAGGTTCCGGGTTCGTGTCCCGGCGGAGTCGCCA	161	3083	18942	18	chr4.trna909-ArgTCT	3E-13
38	TTCAAAGGTTGTGGGTTCGGGTCCCACCAGAGTCGCCA	226	1872	32363	10	chr2.trna1753-ArgTCT	3E-13
38	TTCAAAGGTTGTGGGTTCGTGTCCCACCAGAGTCGCCA	240	1629	21449	16	chr2.trna1753-ArgTCT	3E-13
37	TCAAAGGTTGTGGGTTCGTGTCCCACCAGAGTCGCCA	256	1501	42339	8	chr2.trna1753-ArgTCT	1E-12
37	TCAAAGGTTGTGGGTTCGGGTCCCACCAGAGTCGCCA	298	1182	26202	13	chr2.trna1753-ArgTCT	1E-12
38	TCTGAAGGTCGTGAGTTCGTTCCTCACACGGGGCACCA	352	962	1867	208	chr4.trna1699-MetCAT	7E-13
38	TCTGAAGGTCGTGAGTTCGATCCTCACACGGGGCACCA	1003	211	9770	37	chr4.trna1699-MetCAT	5E-12
39	ATCTGAAGGTCGTGAGTTCGTTCCTCACACGGGGCACCA	841	275	66631	5	chr4.trna1699-MetCAT	2E-13
38	TCTGAGGGTCCAGGGTTCAGGTCCCTGTTCGGGCGCCA	388	811	2981	129	chr7.trna1861-LysTTT	3E-13
39	ATCTGAGGGTCCAGGGTTCAGGTCCCTGTTCGGGCGCCA	1203	163	2153	45	chr7.trna1861-LysTTT	7E-14
38	TCTCAGGGTCGTGGGTTCGTGCCCCACGTTGGGCGCCA	895	249	27650	12	chr7.trna1861-LysCTT	3E-13
38	TCAGAAGATTGCAGGTTCGGGTCCTGCCGCGGTCGCCA	365	881	37663	9	chr7.trna550-ArgTCG	3E-13
38	TCAGAAGATTGCAGGTTCGTGTCCTGCCGCGGTCGCCA	409	731	46728	7	chr7.trna550-ArgTCG	7E-14
36	AGAAGATTGCAGGTTCGGGTCCTGCCGCGGTCGCCA	725	348	98632	3	chr7.trna550-ArgTCG	4E-12
36	AGAAGATTGCAGGTTCGTGTCCTGCCGCGGTCGCCA	793	302	71339	4	chr7.trna550-ArgTCG	4E-12
38	TCTAAAGGTCCCTGGTTCGTTCCCGGGTTTCGGCACCA	543	507	19632	18	chr7.trna1857-PheGAA	3E-13
39	ATCTAAAGGTCCCTGGTTCGTTCCCGGGTTTCGGCACCA	1089	190	14069	25	chr7.trna1857-PheGAA	2E-13
38	CCGAAAGGTTGGTGGTTCGTGCCCACCCAGGGACGCCA	551	500	93495	3	Chr4.trna653-AsnGTT	3E-13

Abundance of testicular tRFs is marked by blue, ovarian by red. Only 3' tRFs containing 3' tRNA end CCA sequence are shown
